# Physiological characteristics during the formation of aromatic components in xylem of *Aquilaria sinensis* induced by exogenous substances

**DOI:** 10.3389/fpls.2024.1461048

**Published:** 2024-11-19

**Authors:** Shengjiang Pang, Zhongguo Li, Qingqing Zhang, Zuwei Tian, Shuokun Deng, Pei Zhang, Shiling Liu, Baoguo Yang, Zaizhi Zhou

**Affiliations:** ^1^ Experimental Center of Tropical Forestry, Chinese Academy of Forestry, Pingxiang, China; ^2^ Research Institute of Tropical Forestry, Chinese Academy of Forestry, Guangzhou, China; ^3^ College of Forestry, Nanjing Forestry University, Nanjing, China; ^4^ Institute of Flower, Fujian Academy of Forestry, Fuzhou, China

**Keywords:** agarwood, aromatic components, physiological characteristics, endogenous hormones, enzyme activity, agarwood-induction technique, induction treatment, *Aquilaria sinensis*

## Abstract

An inductive combination of plant growth regulators, inorganic salts, and fungi is essential for the formation of aromatic components in the xylem of *Aquilaria sinensis*. However, the dynamics of xylem physiology and the relationships between physiological properties and aromatic components after artificial induction remain unclear. In this study, the changes in physiological properties of *A*. *sinensis* xylem during induction were determined and analyzed under four induction treatments and a control group. The defense hormone contents of jasmonic acid, salicylic acid, aminocyclopropane-1-carboxylic acid, and abscisic acid obtained from the four induction treatments increased significantly. However, the concentrations of gibberellin and indoleacetic acid were decreased compared to the control group. An initially upward and then downward trend was observed in the main antioxidant enzyme activities. Additionally, malonaldehyde content decreased obviously, while proline content tended to increase and then decrease as induction continued. The total and soluble sugar content was evidently reduced after treatment, and the soluble sugar content recovered more rapidly with time. Thirty-three aromatic components were identified in all treatments, and the primary aromatic components were terpenes, aromatics and chromones, the relative contents of which varied among treatments. These results provide new insights for optimization and innovation of agarwood induction techniques by exploring the formation of aromatics in the xylem of *A. sinensis* and its physiological responses following induction with exogenous substances (ethephon, NaCl, CaCl_2_ and fungal mixed solution).

## 1 Introduction

*Aquilaria sinensis* (Lour.) Spreng is an extremely valuable tree of the family Thymelaeaceae that is known for its ability to form agarwood in its xylem (Rasool and Mohamed, 2016). It is widely distributed in south China, Laos, Vietnam, Thailand and other southeastern Asian countries (Editorial Committee of Flora of China and Chinese Academy of Sciences, 1999; Institute of Botany and Chinese Academy of Sciences, 2011). *A. sinensis* is also a second-class protected plant in China and was also listed in the Convention on International Trade in Endangered Species of Wild Fauna and Flora (CITES) (https://cites.org/) in 2000. In general, the xylem of *A. sinensis* is bright white and almost devoid of aromatics. However, the dark xylem, which contains aromatic compounds and is known as agarwood, is only formed after the trunk has been damaged by external factors, such as lightning strikes, wind, injuries from fire or artificial cutting, and induction by inorganic salts or fungi (Pang et al., 2022). As a natural perfume, agarwood is commonly used in the production of cosmetics, pharmaceuticals and handcrafted articles. However, natural agarwood takes a long time to form, is produced in very small quantities and is expensive and thus has a level of commercial demand exceeding its availability. The increased cultivation and artificial promotion of agarwood induction techniques are effective ways to protect wild *A. sinensis* and address the scarcity of agarwood resources.

A range of physiological and biochemical reactions are conducted in the xylem of *A. sinensis* after it has been subjected to external stresses. On the one hand, these stresses promote the biosynthesis of jasmonic acid (JA), salicylic acid (SA), 1-aminocyclopropane-1-carboxylic acid (ACC) and abscisic acid (ABA), which are plant defense hormones and important molecular messengers. These hormones can quickly activate the early defense responses of the trunk and produce key primary defense compounds that trigger the biosynthetic pathways of aromatics (Xu et al., 2016; Tan et al., 2019). However, the defensive system of *A. sinensis* is gradually improved and optimized after injury by inhibiting the emission of plant growth hormones, such as gibberellin (GA) and indoleacetic acid (IAA). This response of the trees inputs more energy into the defense system by regulating and controlling growth. In addition, succession of physiological and biochemical changes in the xylem tissues also takes place, including increases in antioxidant enzyme activity, the contents of malondialdehyde (MDA) and free proline (PRO) and carbohydrate metabolism. The *A. sinensis* xylem physio-biochemical properties were previously examined before and after induction with an inorganic salt solution, revealing that superoxide dismutase (SOD) and peroxidase (POD) activities and malondialdehyde (MDA) contents first increased and then decreased with induction time, while starch was rapidly depleted after induction treatment (Zhang et al., 2024a). Regardless of the specific induction stress, plants accumulate malondialdehyde (MDA) and disrupt cellular metabolism in the initial stage as a result of increased membrane lipid peroxidation (Naziz et al., 2019). Plants undergo self-regulation through physiological and biochemical responses at the wound site in response to stress and protect themselves against pathogens. The activities of superoxide dismutase (SOD) and peroxidase (POD) also significantly enhance scavenging of excess reactive oxygen species (ROS), thus ensuring plant survival (Rong et al., 2021). The starch in the *A*. *sinensis* xylem was always consumed, and the soluble sugar content showed an increasing and then decreasing trend with respect to aromatic compound formation (Saw et al., 2014; Liu Y. X. et al., 2022; Zhang et al., 2022b). The plant hormones, enzyme activities and non-structural carbohydrates mentioned above play important roles in the process of agarwood formation. These changes may alleviate biofilm damage and osmotic stress to some extent and further enhance the resistance of plants to abiotic stresses, but their dynamics throughout induction remain unclear (Trovato et al., 2008; Hou et al., 2013; Tamuli et al., 2014; Yu et al., 2015; Zhang et al., 2022a).

Numerous studies have demonstrated that whether the induction stimulus is physical trauma, chemical stimulation or microbial infection, the xylem of appropriately treated *A. sinensis* produces and accumulates secondary metabolites that contain aromatic scents (Ngn et al., 2019; Jamaludin et al., 2020; Sun Y. et al., 2020; Yang et al., 2021; Yan et al., 2024). Many studies have shown that stimulating the xylem of *A. sinensis* with different solutions can readily induce the plant to produce high-quality and large quantities of agarwood compounds. Blanchette and Heuveling reported that the yield of wood that contained aromatics significantly increased after treatment with inorganic salt solutions (NaCl, sodium bisulfate [NaHSO_3_] or iron chloride [FeCl_2_]). In particular, after 12 months of treatment with a solution of NaCl, the sample content of sesquiterpenes reached 1.5%, the level of middle-grade agarwood in the market (Blanchette and Heuveling, 2009). Le et al. conducted a heartwood induction test on *A. crassna* using a mixture of sulfuric acid and sodium methyl bisulfate (CH_3_SO_3_Na) and also found that the resulting samples contained 15.8–20.8% sesquiterpenes, exceeding the aromatic content of the control (Le et al., 2015). Our research group has confirmed that plant growth regulators (including ethephon), inorganic salts and fungi are important inducers that promote the formation of aromatics in the xylem of *A. sinensis* (Chen et al., 2018; Zhao et al., 2024; Pang et al., 2024). Usually, ethephon is hydrolyzed to ethylene, which regulates the synthesis and release of terpenoids in plants. During the process of agarwood formation in *A. sinensis* under different chemical reagents (NaCl, ethephon, and hydrogen peroxide) treatments, the non-structural carbohydrates’ starch content decreased, while the soluble sugar content increased, resulting in increased secondary metabolism in *A. sinensis* and promoting the accumulation of terpenoids and lipids. (Cao et al., 2006; Lei et al., 2011; Song et al., 2020). Meanwhile, the agarwood essential oil yield induced by the ethephon treatment was significantly higher than that induced by sodium chloride, distilled water and wounding treatment (Zhang et al., 2024b).

We recovered and characterized three highly active fungi (*Melanotus flavolivens*, *Rigidoporus vinctus* and *Fusarium solani*) from high-oil-content agarwood and inoculated them into *A. sinensis* using injection methods. Compared to the control group, *A. sinensis* injected with *M. flavolivens* solutions exhibited the broadest discoloration range; the greatest changes were observed in the depletion of starch content and the elevation of soluble sugars, which promoted the accumulation of aromatic components. When the inocula of *R. vinctus* were inoculated into *A. sinensis*, agarwood was found to be induced. The alcohol-soluble extract content of the induced agarwood reached up to 38.9%. The relative percentages of the terpenes in the agarwood essential oil were 22.76%. The proportions of terpenes and chromones in *F. solani* solutions treatments were 62.65% and 70.97%, respectively (Chen et al., 2018; Zhao et al., 2024; Pang et al., 2024). Subsequent research has confirmed that *M. flavolivens*, *R. vinctus* and *F. solani* can induce highyield and high-quality agarwood when used in combination with other chemical reagents. Furthermore, obvious changes in the activities of peroxide dismutase (SOD), catalase (CAT) and peroxidase (POD), as well as the content of MDA, were observed after treatment (Song et al., 2019, 2020). Nevertheless, little is known about the physiological dynamic alterations of aromatics in the xylem of *A. sinensis* under abiotic stress, and this topic merits further exploration.

In this study, a 10-year-old plantation of *A. sinensis* was selected as the study site, and the trees were induced by treating their trunks with different concentrations of the plant growth regulator ethephon, inorganic salts (NaCl and calcium chloride [CaCl_2_]) and a fungal solution, respectively. This study sought to answer the following two questions. (1) What are the trends of xylem physiological properties, including endogenous hormones, antioxidant enzyme activities, MDA, PRO, total sugar and soluble sugar, under different induction treatments? (2) What are the interconnections between the various physiological indicators?

## 2 Materials and methods

### 2.1 Experimental materials

The study was conducted at Anshan Village, Shatang Town, Yulin City, Guangxi Zhuang Autonomous Regin, China (22°53′31″ N, 109°54′17.4″ E), which has a subtropical monsoon climate. The average annual temperature in the region is 22.0°C, the average annual sunshine hours total 1795 hours, and the average annual precipitation is 1650 mm. The climate is mild with synchronicity in abundant precipitation and heat.

A 10-year-old plantation of *Aquilaria sinensis* (Lour.) Spreng was selected for the agarwood induction experiment, and the selected trees were healthy and free of pests and diseases. The average diameter at breast height and tree height were 14.52 ± 0.44 cm and 8.64 ± 0.38 m, respectively. This study was conducted on November 25, 2022. The sampling period from December to May in 2022 and 2023 was characterized by abundant precipitation and adequate sunshine hours with typical seasonal characteristics.

### 2.2 Experimental design and sampling

This study utilized a completely random design with four different treatments, including ethephon (a growth regulatory compound), NaCl, CaCl_2_ and a mixed fungal solution (a mixture of two or three fungal inocula in different volumes). Distilled water was used as the control. The composition of the liquid mixture for each treatment is shown in **Table 1**.

**Table 1:** Plant growth regulators, inorganic salts and fungal mixed solution treatments for induction.

Treatment	Ethephon (%)	NaCl (%)	CaCl_2_ (%)	Fungal solution
T_1_	2.0	2.0	1.5	*Melanotus flavolivens*:*Rigidoporus vinctus* 1:1 solution (*v*:*v*)
T_2_	2.0	1.0	1.0	*Melanotus flavolivens*:*Fusarium solani* 1:1 solution (*v*:*v*)
T_3_	2.0	1.5	0.5	*Rigidoporus vinctus*:*Fusarium solani* 1:1 solution (*v*:*v*)
T_4_	2.0	2.0	1.5	*Melanotus flavolivens:Rigidoporus vinctus*:*Fusarium solani* 1:1:1 solution (*v*:*v*:*v*)
CK	Distilled water treatment			

Three fungal strains were selected as the inoculation materials from the agarwood-forming parts of *A. sinensis* as part of the scientific research project entitled “The Basic Scientific Research Project of the Chinese Academy of Forestry (CAFYBB2022MB004)”. The fungi were identified as *Melanotus flavolivens*, *Rigidoporus vinctus*, and *Fusarium solani*. The three strains were inoculated onto autoclaved potato dextrose agar (PDA) plate medium and incubated at 28°C for 4 days to facilitate strain activation. The requisite quantity of mycelium was inoculated into a 50-L mechanically stirred stainless steel fermenter (Anhui Saideqirui Biotechnology Co., Ltd., Xuancheng, China) containing potato dextrose medium (PD), and incubated at 28°C for 7 days. Subsequently, the fungus culture cultivated in the liquid medium was filtered through a double layer of medical gauze, to prevent the drip catheter from becoming obstructed when draining, at which point it was ready for use as an inoculum.

The cultured fungal inoculum, growth regulators and inorganic salts were combined in accordance with the specified proportions, and all experimental trees were treated with two bags of 500 mL of the mixed solution according to the following treatments: T_1_ treatment, 2.0% ethephon, 2.0% NaCl, 1.5% CaCl_2_, *M. flavolivens*:*R. vinctus* 1:1 culture solution (*v*:*v*); T_2_ treatment, 2.0% ethephon, 1.0% NaCl, 1.0% CaCl_2_, *M. flavolivens:F. solani* 1:1 culture solution (*v*:*v*); T_3_ treatment, 2.0% ethephon, 1.5% NaCl, 0.5% CaCl_2_, *R. vinctus*:*F. solani* 1:1 culture solution (*v*:*v*); T_4_ treatment, 2.0% ethephon, 2.0% NaCl, 1.5% CaCl_2_, *M. flavolivens*:*R. vinctus*:*F. solani* 1:1:1 culture solution (*v*:*v*:*v*); CK, distilled water (Pang et al., 2024).

**Figure 1:**
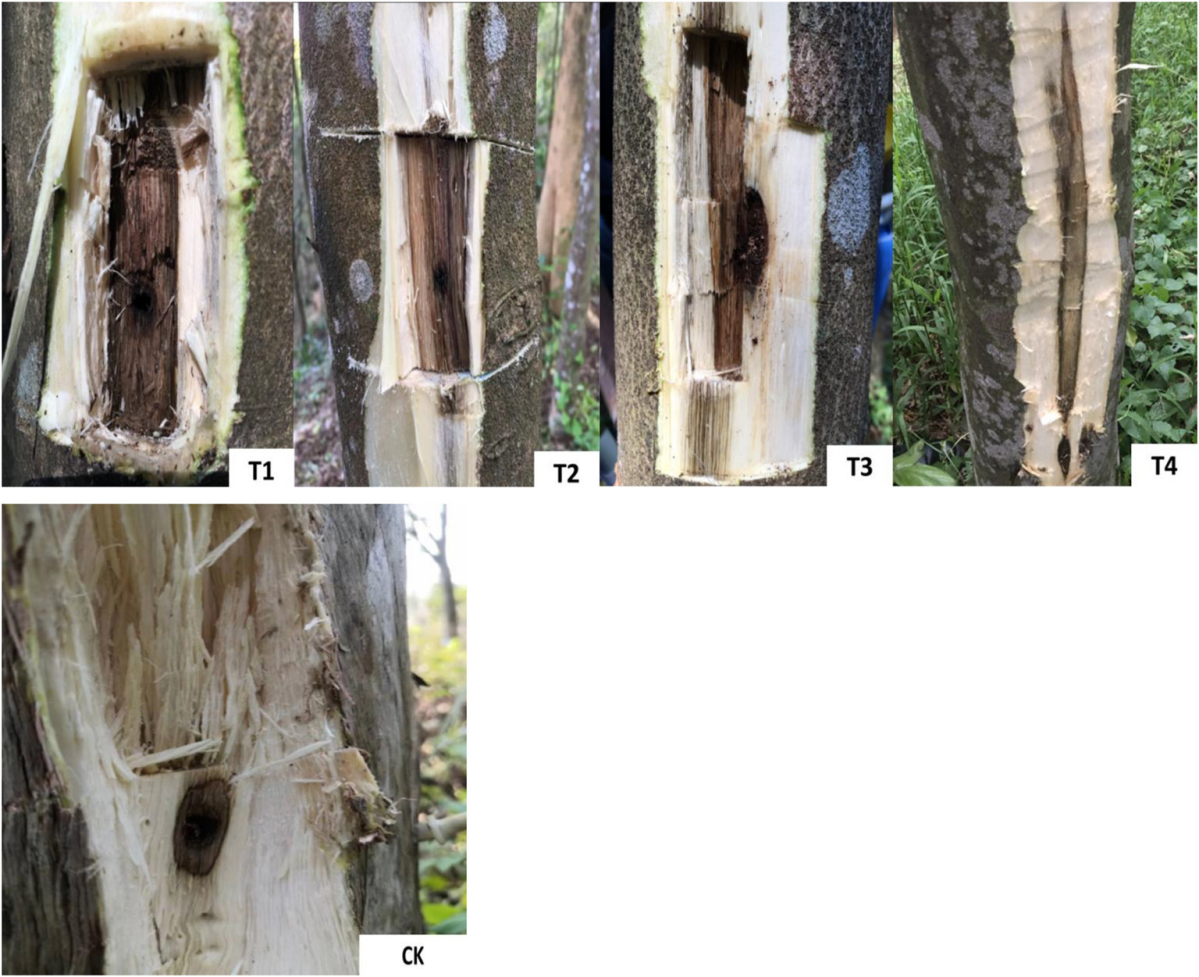
Representative images of the agarwood sampling from the T_1_, T_2_, T_3_, T_4_ and CK groups.T_1_ treatment, 2.0% ethephon, 2.0% NaCl, 1.5% CaCl_2_, *Melanotus flavolivens*:*Rigidoporus vinctus* 1:1solution (*v*:*v*); T_2_ treatment, 2.0% ethephon, 1.0% NaCl, 1.0% CaCl_2_, *M. flavolivens*:*Fusarium solani* 1:1 solution (*v*:*v*); T_3_ treatment, 2.0% ethephon, 1.5% NaCl, 0.5% CaCl_2_, *R. vinctus*:*F. solani* 1:1 solution (*v*:*v*); T_4_ treatment, 2.0% ethephon, 2.0% NaCl, 1.5% CaCl_2_, *M. flavolivens*:*R. vinctus*:*F. solani* 1:1:1 solution (*v*:*v*:*v*); CK, distilled water control treatment.

Four infusion holes were drilled into four sides of the trunks of *A. sinensis* at 60 cm above the ground with a 5-mm drill bit on a sunny day. The infusion method was utilized to inject the induction solution into the trunks. Two 300-mL bags of mixed solution were suspended from each tree. Thirty trees were randomly selected for each treatment with three replicates as described. The samples were collected on days 15, 30, 60, 120 and 180 after induction. Three plants were randomly chosen from each treatment, and wood samples were collected 3–5 cm above the infusion hole of the *A*. *sinensis* trunk (**Figure 1**).

**Figure 2:**
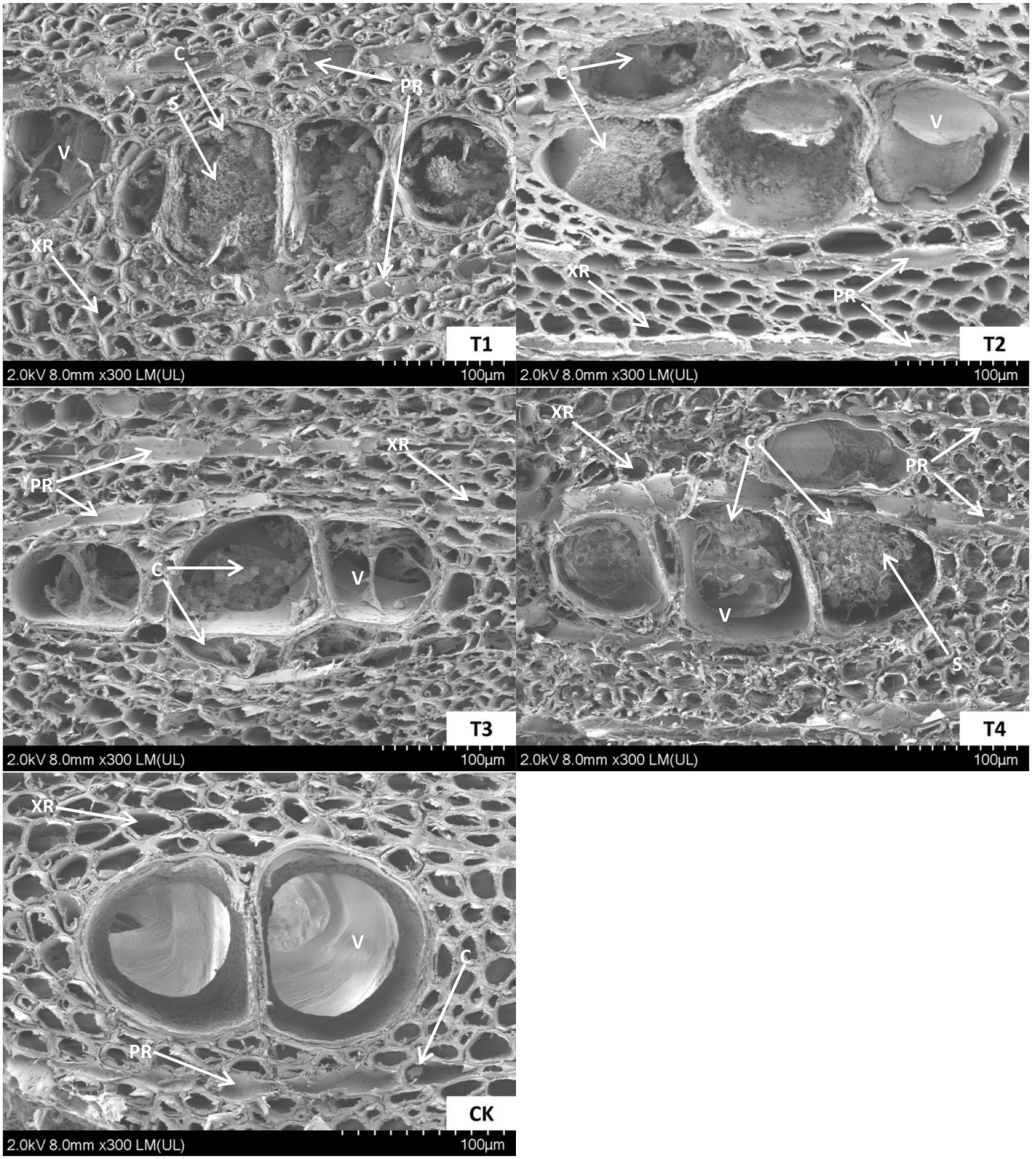
The ultra-microscopic structure of the agarwood samples. Diameter cross-section of agarwood at 180 days of T_1_, T_2_, T_3_, T_4_ and CK induction treatments. T_1_ treatment, 2.0% ethephon, 2.0% NaCl, 1.5% CaCl_2_, *Melanotus flavolivens*:*Rigidoporus vinctus* 1:1 solution (*v*:*v*); T_2_ treatment, 2.0% ethephon, 1.0% NaCl, 1.0% CaCl_2_, *M. flavolivens*:*Fusarium solani* 1:1 solution (*v*:*v*); T_3_ treatment, 2.0% ethephon, 1.5% NaCl, 0.5% CaCl_2_, *R. vinctus*:*F. solani* 1:1 solution (*v*:*v*); T_4_ treatment, 2.0% ethephon, 2.0% NaCl, 1.5% CaCl_2_, *M. flavolivens*:*R. vinctus*:*F. solani* 1:1:1 solution (*v*:*v*:*v*); CK, distilled water control treatment; V, vessel; XR, xylem ray; PR, phloem ray; S, starch grain; C, crystal.

The wood samples fixed with formalin-aceto-alcohol were cut into 2-mm-thick slices and put into a vacuum chamber, where they were sprayed with gold by a JEC-1600 anion sputtering instrument (JEOL Japan Electronics Co., Ltd., Tokyo, Japan) twice for 60 s each time with a 30 mA spraying flow, and then, the samples were fixed under an ultra-high field emission scanning electron microscope (Regulus8100; Hitachi, Ltd., Tokyo, Japan) for observation (**Figure 2**). These samples were wrapped in aluminum foil, placed in liquid nitrogen until completely frozen and eventually stored at -80°C for subsequent measurements.

All collected wood samples have been deposited in the herbarium of the Experimental Center of Tropical Forestry, Chinese Academy of Forestry, and the voucher specimen numbers (T_1_-1-1, T_1_-1-2, T_1_-1-3,…) are provided in Appendix I (**Supplementary Materials Table S1**).

### 2.3 Determination of the physiological indicators

A volume of 5 mL of 80% methanol (Sinopharm Chemical Reagent Co., Ltd., Shanghai, China) and 1.0 mmol/L of 2, 6-di-*tert*-butyl-4-methylphenol (Sinopharm Chemical Reagent Co., Ltd.) cooling buffer were added to 1.0 g of the fresh wood samples. These were ground and extracted, and the homogenate was incubated at 4°C for 4 h and centrifuged at 4000 × *g* for 5 min. A Cytation3 instrument (BioTek, Agilent Technologies, Santa Clara, USA) utilized enzyme linked immunosorbent assay to determine the concentrations of JA, SA, ACC, ABA, GA and IAA according to the manufacturer’s instructions (Wu et al., 1988). The activities of antioxidant enzymes, including SOD, CAT and peroxidase (POD) (Ren et al., 2021), and the content of MDA were determined by the thiobarbituric acid method, and PRO content was determined by acid ninhydrin colorimetry (Magné and Larher, 1992). The contents of total sugar and soluble sugars were determined by anthrone colorimetry (Gao, 2006). The total phenolics were determined by Flynn phenol colorimetry (Galović et al., 2021), and the contents of total terpenes were determined by extraction with organic solvents and UV spectrophotometry (Ghorai et al., 2012).

### 2.4 Determination of aromatics

The wood samples were collected on day 180 after induction. Three plants were randomly chosen from each treatment, and the discolored parts of the trunks that were 3.0–5.0 cm above the drilling position were sampled with a machete, frozen in liquid nitrogen, brought back to the laboratory and air-dried in a dark room. The samples of wood were shredded with a knife, ground into powder with a Panasonic miller (Panasonic, Kadoma, Japan) and then passed through a 40-mesh sieve before assaying their composition. A total of 2.0 g of each sample was weighed and placed in a 50-mL centrifuge tube. A volume of 20 mL of 95% ethanol was then added. The sample solution was treated by ultrasonication (Elmasonic P300H, Elma Ultrasonic, Wetzikon, Switzerland) at 35k Hz and 60°C in a water bath for 2 h. The suspension was obtained by passage through a 0.45-μm filter membrane, and the volumetric mixed solution was brought to 50 mL (Li et al., 2023). A volume of 10 mL of the sample was measured to determine the components using a Temperamental Co-Use Instrument (Agilent 7890B-5977A GC-MS; Agilent Technologies) and HP-5ms column (30 m × 0.25 mm × 0.25 μm; Agilent Technologies). The gas chromatography–mass spectrometry conditions were as follows: start at 70°C and hold for 1 min; increase to 150°C at 10°C/min; 5 min hold; increase at 5°C/min to 260°C; hold for 15 min; inlet temperature 250°C; sample volume 1 μL (shunt ratio 20); ion source temperature 250°C; ionization mode EI electron energy 70 eV; carrier gas He (99.999%); carrier air velocity 0.5 mL/min; mass scanning range 35 to 350 m/z; a solvent delay of 5 min.

To confirm each chromatograph for the qualitative analysis of each component, the retention index of chromatographic peaks and mass spectrometry information were retrieved and compared with the NIST 14 standard mass spectrometry library peak by GC-MS Postrun Analysis software (Yan et al., 2019; Yan et al., 2024). Finally, the relative percentage of each component was calculated using the peak area normalization method.

### 2.5 Statistical analysis

To analyze the dynamic changes of physiological indexes in the xylem of *A. sinensis* under different induction treatments, two-way analysis of variance (ANOVA) and Tukey’s honest significant difference (HSD) test were performed (*p* < 0.05) using SPSS 21.0 (IBM, Inc., Armonk, NY, USA). All the data was first evaluated for the assumptions of their homogeneity of variance and normality of their distribution before statistical analyses. The Mantel test was performed to assess the main drivers that were significantly correlated with aromatic components based on Spearman’s correlated coefficient (*p* < 0.05) using the “vegan” package in Rstudio software (RStudio, Inc., Boston, MA, USA). Graphs were plotted using Origin Pro2023 software (OriginLab Corp., Northampton, MA, USA).

## 3 Results

### 3.1 Changes in the contents of hormones during different induction treatments and times

#### 3.1.1 Defense hormone content

The defense hormone contents showed significant differences among induction treatments (*p* < 0.05, **Supplementary Table S2**). Compared to the CK group, the contents of JA, SA, ACC and ABA were obviously enriched in xylem after the induction treatments, with increases of 31.24–44.85%, 31.73–51.28%, 54.60–70.02% and 28.70–42.59%, respectively. The highest levels of JA, SA and ACC were found in the T_4_ induction treatment, while ABA content was the highest in T_1_, though it did not significantly differ from that in the T_4_ treatment.

The contents of JA, SA and ACC appeared to have a consistent trend of increasing and then decreasing as induction time increased, peaking on day 30 after induction treatment (**Supplementary Table S3**). The minimum levels of JA, SA and ACC were observed on day 180 and were significantly lower than those on day 15. The content of ABA was at its maximum on day 15, but there was no clear trend with increasing induction time.

**Figure 3:**
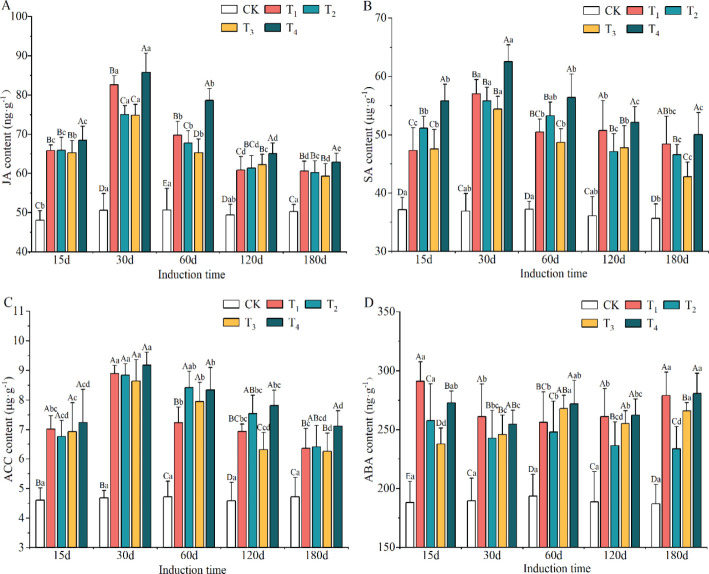
Variation in the content of jasmonic acid (JA) **(A)**, salicylic acid (SA) **(B)**, 1-aminocyclopropane-1-carboxylic acid (ACC) **(C)** and abscisic acid (ABA) **(D)** during different induction treatments and times (n=3). The different letters indicate significant differences at *p* < 0.05 according to Tukey’s honest significant difference test, and the capital and lowercase letters specifically indicate a significant difference at the same time between different treatments and at different times under the same treatment, respectively.

The interaction between induction treatment and induction time on defense hormone contents was visible and significant (**Figures 3A–D**). The contents of JA, SA, ACC and ABA increased significantly in the xylem of *A. sinensis* after the different induction treatments, with higher levels of defense hormone than observed in the CK treatment (*p* < 0.05). The content of ABA in the T_1_ treatment increased the most, at 54.72%, on day 15. JA, SA and ACC each increased the most in the T_4_ treatment at day 30, with increases of 69.42%, 69.47%, and 95.74%, respectively. The content of ABA in the T_1_ treatment still increased by 49.30% by day 180, while that of SA in the T_4_ treatment increased by 50.12%. These findings indicated that *A. sinensis* remained at a high level as a defense response for some period after the induction treatment with different mixed solutions.

#### 3.1.2 Contents of the growth hormones

The levels of GA and IAA after T_1_, T_2_, T_3_ and T_4_ induction treatments declined obviously and were lower than those in the CK group, by 25.48–39.89% and 9.82–14.18%, respectively (**Supplementary Table S4**). The minimum contents of GA and IAA appeared in the T_1_ and T_4_ treatments, respectively. However, the contents of GA and IAA accumulated markedly with increasing induction time (except for IAA on day 180) and the lowest levels were on day 15, which were 15.02% and 4.14% lower than the highest values, respectively.

**Figure 4:**
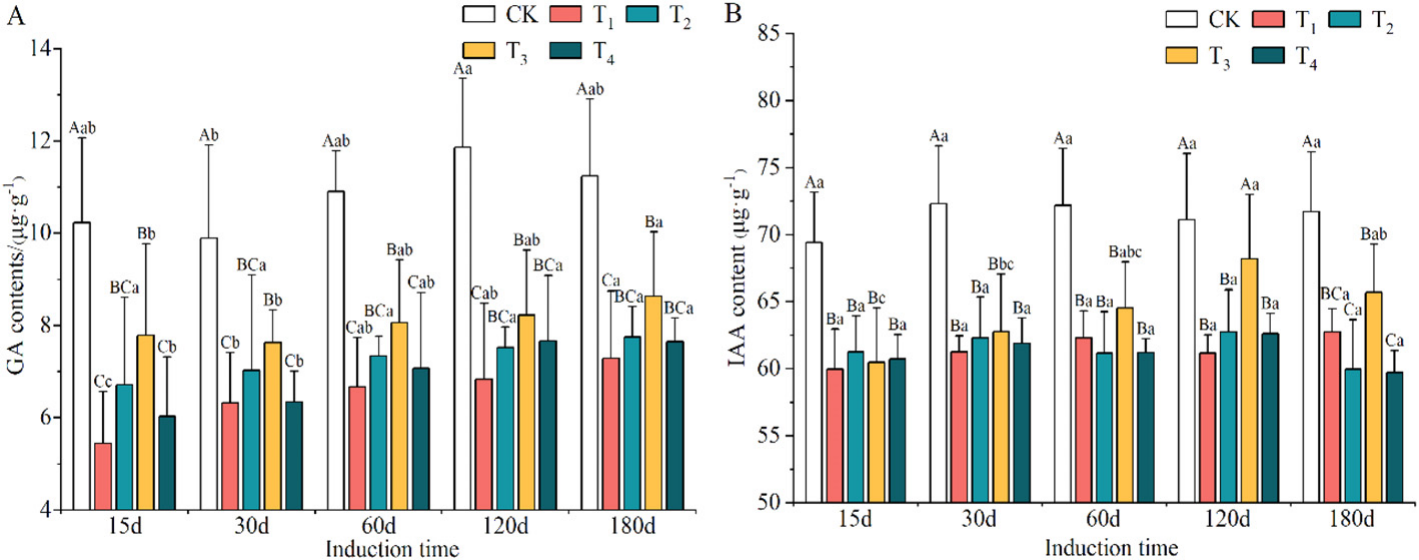
Variation in the contents of gibberellin (GA) **(A)** and indoleacetic acid (IAA) **(B)** in different induction treatments and times. Different capital and lowercase letters denote significant (P < 0.05) differences among the treatments and times in Tukey’s honestly significant difference test and the bar represents the standard deviation (n=3).

As shown in **Figures 4A, B**, the concentrations of GA and IAA in the xylem of *A. sinensis* decreased compared to the control group. The concentration of GA in the T_1_ treatment decreased the most, by 46.73% on day 15, followed by the T_4_ treatment, which had a decrease of 41.06%. By day 180, the decrease in the concentration of GA in all the induction treatments had decreased to between 19.73% and 35.20%. From days 15 to 180, the range of decrease of IAA narrowed to -12.52% to -15.25% in T_1_ and -11.73% to -16.38% in T_2_; it thereafter gradually increased as the defensive ability of the *A. sinensis* tree improved.

### 3.2 Changes in antioxidant enzymes activities during different induction treatments and times

The different induction treatments clearly promoted the activity of antioxidant enzymes compared to CK treatment (**Supplementary Table S5**). The highest SOD and POD activities of T_1_ were significantly different from those of the other treatments and elevated by 7.12–48.67% and 9.00–74.90%, respectively. The CAT activities of T_1_, T_2_, T_3_ and T_4_ were 82.22%, 73.83%, 60.34% and 82.27% higher than that of the CK treatment.

The initially upward and then downward trend was observed in the antioxidant enzyme activities with increasing induction time (**Supplementary Table S6**). The activities of SOD, CAT and POD reached their maximum values on day 30 after induction treatment. The lowest activity levels for SOD and POD occurred on day 180, and that of CAT was on day 15.

**Figure 5:**
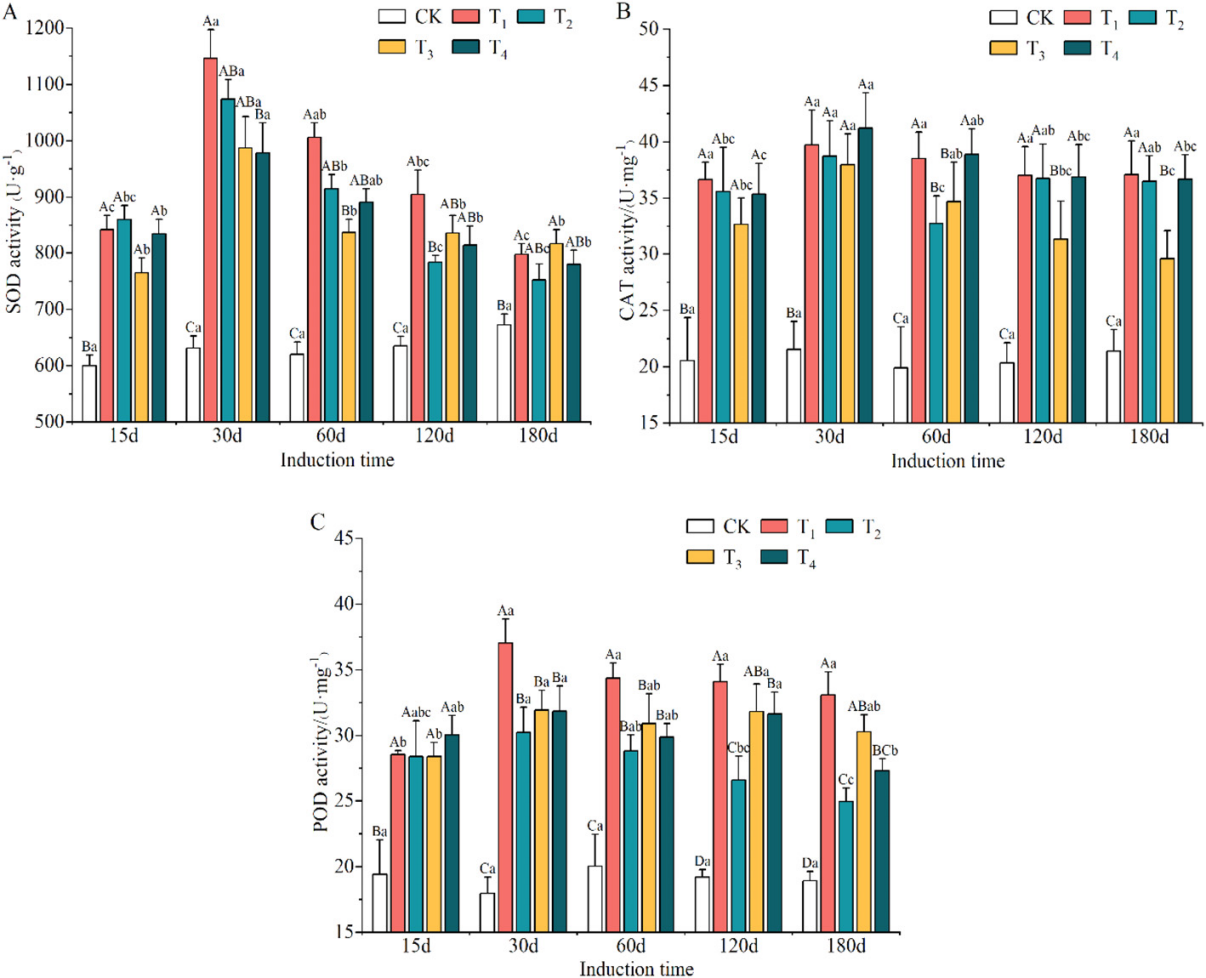
Variation in the activities of SOD **(A)**, CAT **(B)** and POD **(C)** antioxidant enzymes in different induction treatments and times. Different capital and lowercase letters denote significant (P < 0.05) differences among the treatments and times in Tukey’s honestly significant difference test and the bar represents the standard deviation (n=3). SOD, superoxide dismutase; CAT, catalase; POD, peroxidase.

As shown in **Figures 5A–C**, the activities of SOD, CAT and POD in the xylem of *A. sinensis* all increased. The difference between the treatment and CK groups reached a significant level (*p* < 0.05). The activities of SOD and POD in the T_1_ treatment increased the most, by 81.53% and 106.12% at day 30, respectively. The highest increase in the activities of CAT in the T_1_ and T_4_ treatments were 95.23% and 93.32%, respectively, and occurred at day 60. This indicated that *A. sinensis* was subjected to more severe external-stress-induced damage.

### 3.3 Changes in osmoregulatory compounds during different induction treatments and times

An obvious promotion in MDA and PRO contents was observed under the different induction treatments (**Supplementary Table S7**). The greatest increase in MDA and PRO contents was found under the T_4_ treatment, which were increased by 71.07% and 63.73% compared to CK conditions, respectively. Nevertheless, MDA content did not exhibit significant differences among T_1_, T_3_ and T_4_ treatments, and PRO content also lacked significant differences among T_1_, T_2_ and T_3_ treatments. MDA content decreased obviously with increasing induction time, and the minimum (on day 180) was 36.76% lower than that of the maximum (on day 15). As the induction time increased, the content of PRO tended to increase and then decrease, with peaks and troughs occurring on days 30 and 180, respectively.

**Figure 6:**
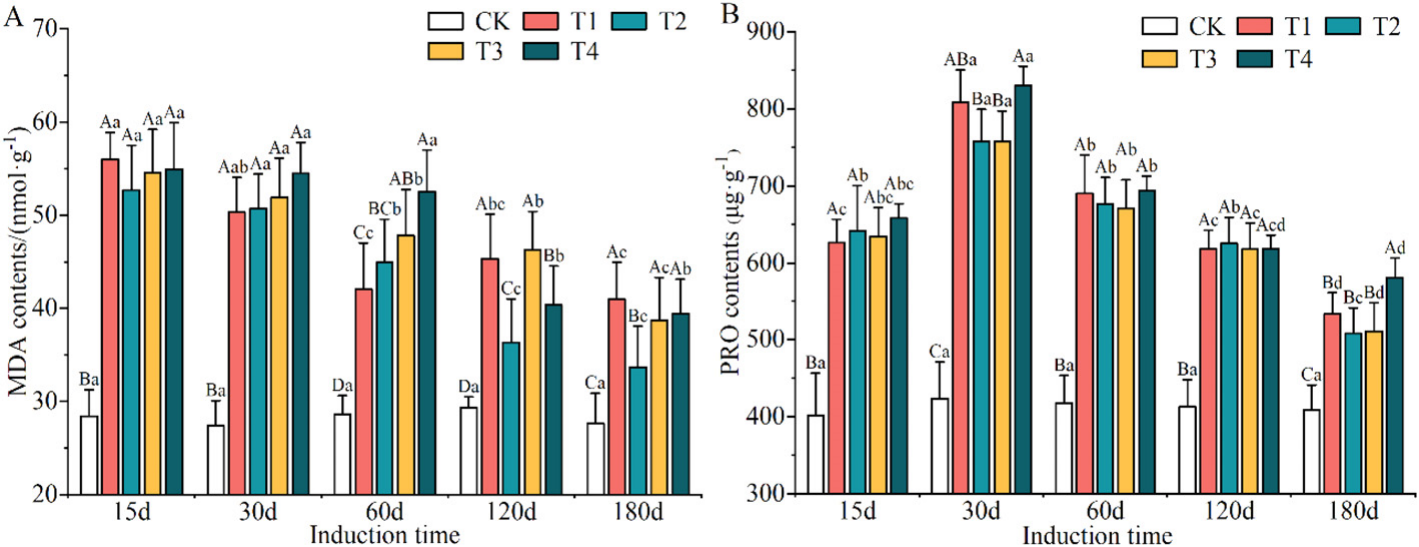
Changes in the contents of MDA **(A)** and PRO **(B)** in the xylem of *Aquilaria sinensis*. Different capital and lowercase letters denote significant (P < 0.05) differences among the treatments and times in Tukey’s honestly significant difference test and the bar represents the standard deviation (n=3). MDA, malondialdehyde; PRO, proline.

With the extension in the time of induction treatment, the contents of MDA (**Figure 6A**) and PRO (**Figure 6B**) in the xylem of *A. sinensis* increased; the difference between treatments and the control group reached a significant level (*p* < 0.05). After each induction treatment, the content of MDA in the xylem of *A. sinensis* reached its maximum on day 15, with an increase that ranged from 38.82% to 97.18%. The increase in the content of MDA decreased by 6.44% to 48.24% by day 180. This indicates that the injection of the mixed solution into the *A. sinensis* xylem during the initial stage of stress increased the content of MDA and significantly inhibited its growth.

The content of PRO in the xylem of *A. sinensis* increased initially followed by a subsequent decrease over time. After each induction treatment, the content of PRO reached its maximum on day 30, with an increase that ranged from 71.54% to 95.97%. The content of PRO also has increased more slowly by day 180, with an increase that ranged from 19.55% to 41.95%. This indicates that the accumulation of free PRO in the xylem of *A. sinensis* gradually decreased, which reduced autotoxic damage to the tree by maintaining osmotic regulation early on and demonstrated that it was highly adaptable to adverse conditions.

### 3.4 Contents of total sugar and soluble sugars

The total and soluble sugar contents were evidently reduced after induction treatment (**Supplementary Table S8**), suggesting that these were consumed when the trees were subjected to external stresses; among the different induction treatments they were 16.81–17.13% and 17.93–21.85% lower than in CK conditions. The total sugar content decreased and then increased as induction time increased, while the soluble sugar content exhibited no clear trend.

**Figure 7:**
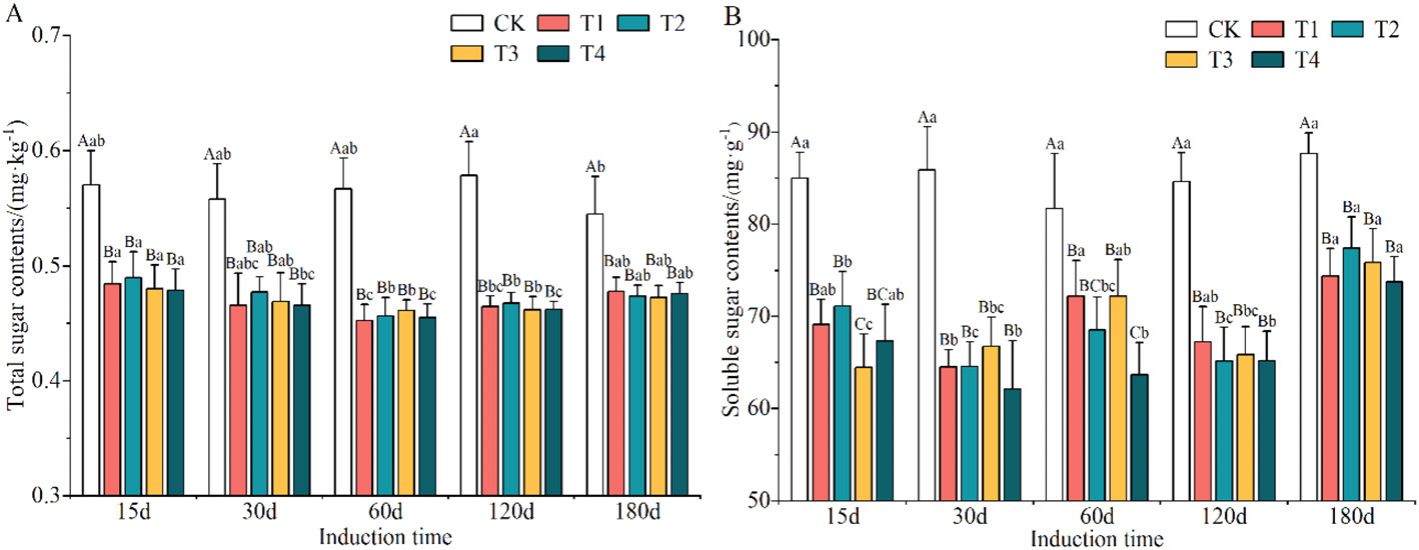
Variation in the contents of total sugar **(A)** and soluble sugar **(B)** in the xylem of *Aquilaria sinensis*. Different capital and lowercase letters denote significant (P < 0.05) differences among the treatments and times in Tukey’s honestly significant difference test and the bar represents the standard deviation (n=3).

The changes in the contents of total sugar and soluble sugars in the xylem of *A. sinensis* after the trees had been subjected to various induction treatments are shown in **Figures 7A, B**. There were significant differences in the contents of both the total sugar and soluble sugars in the xylem in *A. sinensis* compared with the CK group (*p* < 0.05). Over time, the content of total sugar showed a wave-like trend and that of the soluble sugars decreased gradually and then increased again. The content of total sugar in most treatments reached its lowest point on day 60, with a decrease that ranged from 19.16% to 22.24%. The content of soluble sugars in the xylem of *A. sinensis* recovered by day 180 in most of the treatments.

Overall, the sugars in the xylem of *A. sinensis* were metabolized, and the induction treatment with a higher concentration of inorganic salts in the mixed solution resulted in the metabolism of more sugars relative to the lower concentration, indicating that the former significantly accelerated the metabolism of sugar in the xylem of *A. sinensis*. The changes in the contents of sugar reflected a strategy used by the *A. sinensis* trees to adapt to stress.

### 3.5 Relative contents of aromatics

After four induction treatments, 33 aromatic components were identified in the xylem of *A. sinensis*. These major components included terpenes, aromatics and chromones (**Table 2**; **Supplementary Figure S1**). There were 24, 13, 13 and 15 detected aromatic components in agarwood induced by the T_1_, T_2_, T_3_ and T_4_ treatments, respectively. The T_1_ treatment contained the greatest number of major aromatic components, 34.60% of detected aromatics, followed by the T_3_ (20.09%) and T_4_ (19.13%) treatments, while the T_2_ treatment contained the fewest detected aromatics (8.19%).

**Table 2:** Major aromatics of *Aquilaria sinensis* under different treatments used to induce agarwood formation.

No.	Components	Molecular Formula	Odor*	Relative content (%)				
				T_1_	T_2_	T_3_	T_4_	CK
1	α-Guaiene^△^	C_15_H_24_	Sweet, woody, creamy, pepper flavored	0.34	0.26			–
2	Valerenol^△^	C_15_H_26_O	Woody, cream and muskiness	0.38	0.31	0.34	0.19	–
3	α-Santalol^△^	C_15_H_24_O	Soft sandalwood	0.72	0.35		0.34	–
4	β-Myrcene^△^	C_10_H_16_	Orange-like and balsam aromas	0.53	–	–	–	–
5	Eremophilone^△^	C_15_H_22_O	Fumigate, pepper, leather, vanilla, and an earthy odor	–	0.23	–	–	–
6	Benzaldehyde^□^	C_7_H_6_O	Almond, cherry, and nutty aromas	0.33	0.18	0.43	0.26	
7	Longipinocarvone^△^	C_10_H_14_O	Mint and licorice odors	–	–	0.24		–
8	Benzylacetone^□^	C_10_H_12_O	Fruity, floral, medicinal	0.24	0.36	0.33	0.29	–
9	Longifolene^△^	C_15_H_24_	sweet woody rose medical fir needle	1.26	1.33	3.11	0.88	0.06
10	Aromandendrene^△^	C_15_H_24_	Orange-like aromas	0.14	0.17	–	0.41	–
11	5,11-Guaiadiene^△^	C_15_H_24_	Moldy odor	0.24	–	–	–	–
12	Alloaromadendrene^△^	C_15_H_24_	Sweet, woody	0.38	–	–	0.17	–
13	β-Humulene^△^	C_14_H_22_	Mint, clove, and turpentine	0.33	–	–	0.28	–
14	Valerena-4,7(11)-diene^△^	C_15_H_24_	Lilac, woody, and spicy aromas	0.36	–	–	–	–
15	Aromadendrene oxide-(2)^△^	C_15_H_24_O	Orange-like aromas	0.24	0.22	–	–	–
16	Spathulenol^△^	C_15_H_24_O	Earthy herbal fruity		1.55	–	–	–
17	Squalene^△^	C_30_H_50_	Slight pleasant odor	–	–	0.28	–	–
18	Longifolenaldehyde^△^	C_15_H_24_O	Leaf, fruity, vegetable aromas	–	0.20	–	0.19	–
19	β-Vatirenene^△^	C_15_H_22_	Orange-like aromas	0.15		0.53	0.46	–
20	Hinesol^△^	C_13_H_10_O	Medicinal	0.22	0.34		0.18	–
21	Agarospirol^△^	C_15_H_26_O	Woody, nutty aromas, burnt	0.22	1.64	0.51	0.31	0.07
22	Oleic acid	C_18_H_34_O_2_	Fatty	0.13	–	–	–	0.12
23	Valerenic acid^△^	C_15_H_22_O_2_	Disagreeable odor	0.22	–	0.79	1.64	–
24	2-(2-phenylethyl)chromone^○^	C_17_H_14_O_2_	Almond, woody and nutty aromas	13.79	1.04	1.60	3.08	–
25	2-[2-(4-methoxyphenyl)ethyl]chromone^○^	C_18_H_14_O_3_	Almond, woody and nutty aromas	–	–	–	2.52	–
26	6-methoxyl-2-(2-phenylethyl)chromone^○^	C_18_H_16_O_3_	Almond, woody and nutty aromas	4.49	–	4.86	0.66	–
27	6-methoxyl-2-[2-(4′-methoxyphenyl)ethyl]chromone^○^	C_19_H_17_O_3_	Almond, woody and nutty aromas	2.25	–	1.32	0.14	–
28	6,8-Dimethoxy-2-(2-phenylethyl)chromone^○^	C_19_H_20_O_3_	Almond, woody and nutty aromas	–	–	–	0.17	–
29	2-(4-Methoxyphenethyl)chromene^○^	C_18_H_15_O_4_	Almond, woody and nutty aromas	1.00	–	–	–	–
30	6-Methoxy-2-(4-methoxyphenyl)ethyl]chromone^○^	C_19_H_18_O_4_	Almond, woody and nutty aromas	0.22	–	–	–	–
31	6,7-Dimethoxy-2-phenethylchromone^○^	C_19_H_18_O_4_	Almond, woody and nutty aromas	6.46	–	4.21	4.22	–
32	6,7-Dimethoxy-2-[2-(4′-methoxyphenyl)ethyl]chromone^○^	C_20_H_20_O_5_	Almond, woody and nutty aromas	1.09	–	1.55	2.29	–
33	Stigmasterol	C_29_H_48_O	Sterol odor	0.17	–	0.27	0.35	–
The relative content of terpene compounds (%)				4.73	6.61	5.80	5.05	0.13
The relative content of aromatic compounds (%)				0.57	0.54	0.76	0.55	–
The relative content of chromone compounds (%)				29.30**a**	1.04c	13.53b	13.53b	–
The total relative content of aromatic components (%)				34.60	8.19	20.09	19.13	–

△, Terpene compound; □, aromatic compound; ○, chromone compound. Different lowercase letters indicate significant differences among treatments according to Tukey’s honest significant difference test (p < 0.05). *Aromatic odor database, https://www.cas.org/zh-hans/support/documentation/cas-databases.

Chromones and terpenes were the primary aromatics of agarwood in this study, and their relative contents also varied among induction treatments. Under the T_1_ treatment, the relative content of chromones was 29.30%, which was significantly higher than that of the other treatments. The T_2_ treatment contained the highest relative content of terpenes, while those of the chromones were at a relatively lower level. The primary terpenes in the plants from the T_1_ treatment included santalol (C_15_H_24_O, 0.72%) and β-myrcene (C_10_H_16_, 0.53%), while those in the T_2_ treatment included longifolene (C_15_H_24_, 1.33%), spathulenol (C_15_H_24_O, 1.55%) and agarospirol (C_15_H_26_O, 1.64%). Plants in the T_3_ treatment contained longifolene (C_15_H_24_, 3.11%), valerenic acid (C_15_H_22_O_2_, 0.79%), valencene (C_15_H_24_, 0.53%) and agarospirol (C_15_H_26_O, 0.51%), Finally, plants from the T_4_ treatment contained valerenic acid (C_15_H_22_O_2_, 1.64%), longifolene (C_15_H_24_, 0.88%) and valencene (C_15_H_24_, 0.46%).

### 3.6 Relation between the aromatics and physiological properties

**Figure 8:**
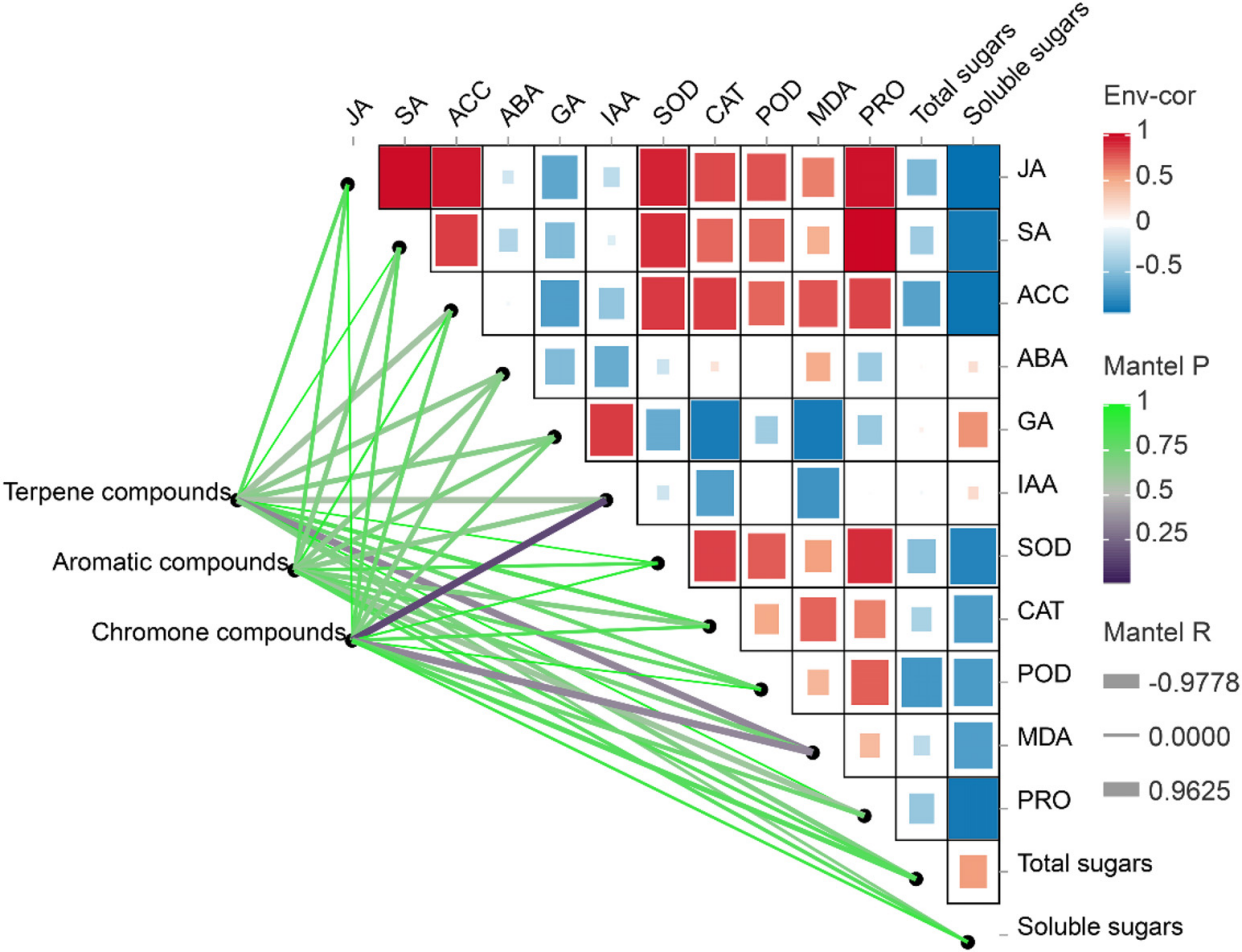
Partial mantel tests between the aromatic components and physiological indices. The heatmap shows the pairwise correlations between physiological indices. The lines denote mantel test results, with the line width represents Mantel’s r statistic, and the color represents Pearson’s correlation coefficient. ABA, abscisic acid; ACC, 1-aminocyclopropane-1-carboxylate; CAT, catalase; GA, gibberellin; IAA, indoleacetic acid; JA, jasmonic acid; MDA, malondialdehyde; POD, peroxidase; PRO, proline; SOD, superoxide dismutase.

A Mantel analysis between the aromatics and physiological properties showed that the terpenes and chromones were significantly negatively correlated with the content of MDA (*p* < 0.05) (**Figure 8**). An obvious positive correlation was observed between the contents of the chromone compounds and IAA. However, no significant correlation was observed between the presence of aromatic compounds and physiological properties (*p* > 0.05).

A correlation heatmap among the physiological indices also indicated that there was a significant positive correlation among JA, SA, ACC, SOD, CAT, MDA and PRO activities/contents, and most of the indicators (**Figure 8**). Most of these indicators were significantly negatively correlated with the contents of GA, IAA, total sugar and soluble sugars. The soluble sugars, in particular, were significantly negatively correlated with JA, SA, ACC, MDA and PRO activities/contents.

## 4 Discussion

### 4.1 Effect of different induction treatments on the contents of endogenous hormones

In this study, the application of inducers increased the contents of JA, SA, ACC and ABA in the xylem of *A. sinensis* compared with the CK group (**Figures 3A–D**), which suggested that the exogenous mixed solution simulated the plant’s defense system. Previous research revealed that JA and SA are key defense hormones whose biosynthesis in plants can be accelerated under external stresses; moreover, they promote the accumulation of agarwood sesquiterpenes (Liu et al., 2015). As a potential novel phytohormone and signaling molecule, ACC plays an important role in the stress response of plants, and it inhibits plant growth in a manner similar to that of ABA. Elevated levels of both hormones not only protect a tree from injury to some extent, but they also promote the accumulation of metabolites by rapidly reducing the viability of cells (Okudera and Ito, 2009; Liu J. et al., 2021). The increase in these defense hormones tended to decrease with induction time in this study. This variation could be explained by the fact that exogenous abiotic stresses diminish the degree of damage to the tree over time (Li et al., 2020).

The contents of growth hormones, such as IAA and GA, clearly decreased during the initial stage of induction and was lower than those in the control, and their levels tended to increase during the later stage of induction treatments (**Figures 4A, B**). Plants are more sensitive to abiotic stress during the early stage of induction, and more materials are metabolized to synthesize defensive substances instead of growth hormones. As the defense system is enhanced and a tree recovers from injury in the later stage, the ability to produce growth hormones is gradually restored. This is similar to the adaptive changes in endogenous hormones in *Dalbergia odorifera* (Cui et al., 2019; Cui et al., 2021) and gingko (*Ginkgo biloba*) (Xu et al., 2009) under external stress. Thus, after treatment with a mixed solution used for induction, the endogenous hormones in *A. sinensis* played a role in signal transmission (Ghorai et al., 2012), activated the function of defense responses in the tree and regulated its resistance and development of growth (Hou et al., 2013; Yu et al., 2015; Zhang et al., 2020; Aerts et al., 2021).

The correlation analyses in this study also showed that the contents of JA, SA, ACC and ABA were significantly negatively correlated with those of GA and IAA, and they were also closely related to the content of sugar in the xylem tissues (**Figure 8**). These results revealed that there is an antagonistic relationship between defense and growth hormones, which together regulate the trade-off between defense and growth during agarwood aromatics formation. It was also observed that vessels were blocked by the tyloses after the induction treatment, which disappeared gradually over time. The disappearance of tyloses is postulated to enhance water transport, thereby contributing to plant growth restoration (Oyarce and Gurovich, 2011). The formation and elimination of tyloses could be attributed to the tradeoff between growth and defense during the formation of aromatic components in *A. sinensis* (Kashyap et al., 2021; Zhang et al., 2022a). Future research should focus on the formation of these aromatic components in *A. sinensis* and explore the complex interactions among the different hormones. It is noteworthy that agarwood production is currently limited, but the use of plant growth regulators or inhibitors of hormone synthesis to balance growth and defense of *A. sinensis* could significantly increase production. This approach has immense commercial potential and practical significance.

### 4.2 Effect of different induction treatments on the activities of enzymes and osmoregulatory compounds

This study showed that there were higher activities of SOD, POD and CAT in the xylem of *A. sinensis* after different induction treatments compared with the control (**Figures 5A–C**). This is consistent with previous findings that the concentrations of antioxidant enzymes in *A. sinensis* (Xu et al., 2016; Song et al., 2019; Sun P. et al., 2020) and hybrid poplar (*Populus simonii* × *P. pyramidalis* ‘Opera 8277’ and *P. tremula* × *P. alba*) (An et al., 2009; Ahmed et al., 2021) increased after exogenous stress treatments. It has been demonstrated that these compounds jointly participate in regulating the metabolism of reactive oxygen species, the degree of cell membrane lipid peroxidation and the protection of trees from external stresses (Farooq et al., 2012; Liu B. et al., 2021). The contents of MDA and PRO in the xylem of *A. sinensis* decreased over time, which may be related to the amelioration of cell membrane damage during the later stages of treatment with the mixed solution. Thus, the permeability and integrity of the cell membrane were effectively managed, which further improved the resistance of trees (Zhang et al., 2014). Clear positive correlations were exhibited between the activities of SOD, POD, and CAT and the contents of MDA and free PRO, and they were also positively correlated with JA, SA and ACC (**Figure 8**). These indicators reflect the self-regulatory ability of plants in stress environments and further suggest that there is a synergistic effect between them.

The formation of aromatic components in *A. sinensis* may be linked to the increase of antioxidant enzyme activities and defense substances (Zhang et al., 2022a; Zhang et al., 2022b; Zhang et al., 2024a). This correlation could be attributed to the induction treatments stimulating the biosynthesis of JA, SA and ACC. These compounds play a pivotal role in activating signal transduction pathways and modulating the plant’s defense responses. As these hormones accumulate within a plant, they trigger the activation of defense genes encoding protease inhibitors, selenoproteins and enzymes involved in secondary metabolism (Savatin et al., 2014). Maintaining ROS homeostasis is essential for plant health, and the scavenging of excess ROS is facilitated by antioxidant enzymes, including SOD and POD. Additionally, secondary metabolites, including flavonoids, terpenoids and phenols, as well as other antioxidants, also contribute to reactive oxygen species scavenging. This not only protects plants from different stresses but also improves their adaptability (Belete, 2018; Gao et al., 2020; Wei et al., 2020; Rong et al., 2021). Previous studies have observed differences in antioxidant enzyme activities, agarwood production and aromatic component composition during the formation of aromatic components in *A*. *sinensis* under a series of induction treatments and among seed sources. For instance, Pripdeevech et al. identified seventy-four volatile substances from *A. malaccensis*, *A. crassna* and *A. subintegra* using gas chromatography-olfactometry (Pripdeevech et al., 2011). These substances, including chromones, β-agarofuran, benzylacetone, furan-2-carbaldehyde and benzaldehyde, are characteristic of the agarwood aroma. Zhang et al. conducted a comprehensive study evaluating the effects of four different treatments, i.e., fire drill treatment, fire drill + brine treatment, cold drill treatment and cold drill + brine treatment, on the aromatic components and metabolomic profiles of *A. sinensis* (Zhang et al., 2011). A total of 712 metabolites composed mostly of alkaloids, amino acids and their derivatives, flavonoids, lipids, phenolic acids, organic acids, nucleotides and their derivatives and terpenoids were detected. The differentially accumulated metabolites were enriched for involvement in flavonoid/flavone and flavonol biosynthesis, sesquiterpenoid and triterpenoid biosynthesis.

### 4.3 Effect of different induction treatments on the contents of various compounds

The sugars produced by photosynthesis play an important role in maintaining the metabolism and physiological functions of biological organisms (Savitch et al., 2010). In this study, the tendency of the contents of total sugars and soluble sugars to decrease in the xylem of *A. sinensis* was apparent during the early stage of the induction treatment (**Figures 7A, B**), and these contents were significantly lower than those of the control. This means that the plants metabolized some of their sugars while resisting stress, and we also found that more sugars were metabolized in the treatment with higher concentrations of the inorganic salt mixed solution than in the treatment with lower concentrations. Some researchers have concluded that sugars promote osmotic adaptation under various abiotic stresses by regulating photosynthesis, carbohydrate metabolism, osmotic homeostasis, protein synthesis, and gene expression to protect cell membranes and cellular proteins from stress (Farhad et al., 2011; Noctor et al., 2015). In addition to their protective role, sugars are also fuel for plant growth and development, are precursors of secondary metabolites and signaling molecules and regulate plant responses to the environment through their molecular functions (Zeid, 2009; Rezaul et al., 2019). Liu et al. examined the structural characteristics of *A. sinensis* and their association with agarwood formation (Liu et al., 2019). They found that interxylary phloem, along with xylem rays, is the main tissue that contains living parenchyma cells in the wood of healthy *A. sinensis*. The main energy reserve present in these parenchyma cells exists in the form of starch grains. After Agar-Wit treatment, these starch grains undergo a series of transformations ultimately transforming into agarwood resin. Non-starch polysaccharides and phenols serve as intermediate products in the process of agarwood resin formation. The resin forms initially and accumulates mainly within the parenchyma cells of the interxylary phloem and xylem rays. These results indicated that not only are the interxylary phloem and xylem rays the initial sites of agarwood resin formation but that they also serve as the main aromatic compound accumulation site. Furthermore, the stored starch grains might serve as the material underlying the formation of the resin. This also indicates that sugar metabolism in the xylem of *A. sinensis* is not only involved in maintaining cellular osmotic regulation but is also directly or indirectly involved in the biosynthesis of secondary metabolites, which is an adaptive response to stress in *A. sinensis*.

In this study, 180 days of treatment with different types of inducers resulted in significant changes in the interaction among xylem tissue structure, physiological characteristics and aromatics during the formation of woody aromatic components in *A. sinensis*. The relative contents of terpenes, aromatics and chromones in the xylem of *A*. *sinensis* were clearly enhanced after the induction treatments with the fungal mixed solutions compared with the control. Terpenes, aromatics and chromones are the key secondary metabolites that plants use to defend against free radicals (Takemoto et al., 2008; Liu et al., 2024). Meanwhile, terpenes and chromones are among the most important characteristic aromatic components of agarwood (Shivanand et al., 2022). Caryophyllene and 2-(2-phenylethyl)chromone isolated from agarwood have antibacterial properties (Dahham et al., 2015; Lei et al., 2018). Similar results were obtained by Ito et al., who concluded that the increase in terpene content is an important physiological response for *A. sinensis* to initiate defense and resist damaging stress (Ito et al., 2005). This indicates that under the long-term impact of the fungal mixed solution, *A*. *sinensis* produces secondary metabolites, including aromatic components, which can enhance its stress resistance and further verify the close relationship between the fungi and agarwood formation.

Mixed fungal inocula can act as elicitors of defense responses in *A. sinensis* (Zhao et al., 2024), and plant hormones, such as ethylene and JA, play a key signaling role. Studies have also shown that multiple fungi can promote the biosynthesis of terpenes through the mevalonic acid (MVA) pathway by modulating the concentrations of plant hormones (Kou et al., 2021; Gastaldo et al., 2019). One of the biosynthetic pathways of chromones is hypothesized to involve the acetate metabolism pathway of the condensation of five malonyl coenzyme A molecules, the biosynthesis of 5, 7-dihydroxy-4-methylchromone by polyketide synthase and the production of 2-(2-phenylethyl)chromone under the catalysis of cyclooxygenase, which catalyzes the biochemical reaction of the chromone matrices using hydroxylases or *O*-methyltransferases, among other enzymes, to form chromones (Amen et al., 2021). Secondly, 2-(2-phenylethyl)chromone was produced by the decarboxylation of malonyl coenzyme A, which was catalyzed by polyketide synthase to produce acetyl coenzyme A as an initial substrate, and hydroxylase and *O*-methyltransferase then catalyzed the production of chromones (Liao et al., 2018).

The highest levels of the total relative content of aromatics were observed in the T_1_ treatment, which indicated that the stress responses of *A*. *sinensis* under the T_1_ treatment were more intense than those in the other treatments, which thus particularly favored the formation of aromatics. This may be owing to the differential expression of genes related to the biosynthesis of secondary metabolites during the stress reactions in plants. In a study on the interaction between *A*. *malaccensis* and a fungal mixed solution, the lipoxygenase (LOX) pathway in the tree was also activated by the fungi and induced the production of free fatty acids (Sen et al., 2017). The antioxidant enzymatic activity of *A*. *sinensis* was enhanced during the early stage of fungal colonization, which triggered a cascade reaction of fatty acid oxidation. This process resulted in a significant increase in the concentrations of JA, SA and ACC, which induced the expression of sesquiterpene synthase genes and polyketide synthase genes, leading to the continuous accumulation of terpenes and chromones and ultimately the formation of agarwood (Küpper et al., 2009; Xu et al., 2017; Lv et al., 2019; Zhang et al., 2022b). The infection of *A*. *sinensis* callus tissue with *Phaeoacremonium rubrigenum* induced a significant upregulation of farnesyl diphosphate synthase (FPS) gene expression, Ses TPS1 and Ses TPS2, which confirmed that the fungi primarily induced the biosynthesis of terpenes through the MVA pathway (Huang et al., 2023). During the biosynthesis of terpenes, significant changes were observed in the levels of protein phosphorylation in 52.9% of the transcription factors (TFs), which included phosphorylation of MYB (V-myb avian myeloblastosis viral oncogene homolog), basic leucine zipper (bZIP) and WRKY TFs that were closely related to enzyme activity in the terpene biosynthetic pathway. During the fungal colonization of *A*. *sinensis*, the accumulation of chromones is promoted by regulating the expression of chalcone synthase (CHS). Under stimulation by *F*. *solani*, the expression of CHS in *A. sinensis* was upregulated after 2 months, and the accumulation of chromones was detected, thus, indicating that this type of component is related to the upregulation of CHS expression in *A. sinensis* after fungal stimulation (Chen et al., 2017).

In summary, there were important interactions between exogenous substances, endogenous hormones, oxidative metabolic pathways and stress-resistance compounds during the formation of aromatics in the xylem of *A. sinensis*. When the plants were subjected to induction with mixed fungal inocula, there was an increase in the biosynthesis of defense hormones rather than growth hormones made possible by the metabolism of more sugars. The activities of protective enzymes were also enhanced to scavenge oxidative free radicals. The osmotic function of cells was also protected by increasing the contents of MDA and PRO. However, the formation of agarwood is a long and complex physiological and biochemical process, is regulated by various factors and involves multiple secondary metabolic pathways. In addition, future research should focus more on the potential molecular mechanisms of agarwood formation, such as the interaction involving functional genes between exogenous substances, agarwood and the key aromatic components.

According to the results of the present study, when exogenous substances are used to induce agarwood formation, induction treatments should include the injection of an appropriate volume of some plant growth regulators, inorganic salts, and fungal mixed solutions into *A*. *sinensis* according to the size of the tree to maintain a higher level of stress to induce the production of high-quality agarwood.

## 5 Conclusions

This research showed that the induction of agarwood using ethephon, salt and fungal mixed solutions could induce a range of changes in the physiological characteristics of the *A. sinensis* xylem. In particular, the contents of defense hormone contents, resistant enzyme activities and osmoregulatory chemical concentrations increased rapidly during the early stage of induction, which affected the biosynthesis of growth hormones. Over time, the increase in these compounds weakened during the later stage of treatment, with saccharides utilized as the primary energy source for agarwood formation.

Our research suggests that the application of the T_1_ induction treatment, i.e., a mixed 1:1 solution (*v*:*v*) of *Melanotus flavolivens* and *Rigidoporus vinctus* inocula with 2.0% ethephon, 2.0% NaCl and 1.5% CaCl_2_, is a particularly suitable induction treatment that facilitates the formation and accumulation of aromatics in the xylem of *A. sinensis*.

## Data Availability

The original contributions presented in the study are included in the article/**Supplementary Material**. Further inquiries can be directed to the corresponding author.
