# Supplementary material for: Physiological characteristics during the formation of aromatic components in xylem of Aquilaria sinensis induced by exogenous substances
Source: Front Plant Sci. 2024 Nov 19;15:1461048. doi: 10.3389/fpls.2024.1461048 (PMC11612829; doi:10.3389/fpls.2024.1461048)

Supplementary Materials

Table S1 Sample number and time of sampling in xylem for *Aquilaria sinensis* under different induction treatments.

| Sample numbers | Collection date | Sample numbers | Collection date | Sample numbers | Collection date | Sample numbers | Collection date | Sample numbers | Collection date |
| --- | --- | --- | --- | --- | --- | --- | --- | --- | --- |
| T_1_-1-1 | 20221210 | T_1_-2-1 | 20221225 | T_1_-3-1 | 20230125 | T_1_-4-1 | 20230325 | T_1_-5-1 | 20230525 |
| T_1_-1-2 | 20221210 | T_1_-2-2 | 20221225 | T_1_-3-2 | 20230125 | T_1_-4-2 | 20230325 | T_1_-5-2 | 20230525 |
| T_1_-1-3 | 20221210 | T_1_-2-3 | 20221225 | T_1_-3-3 | 20230125 | T_1_-4-3 | 20230325 | T_1_-5-3 | 20230525 |
| T_2_-1-1 | 20221210 | T_2_-2-1 | 20221225 | T_2_-3-1 | 20230125 | T_2_-4-1 | 20230325 | T_2_-5-1 | 20230525 |
| T_2_-1-2 | 20221210 | T_2_-2-2 | 20221225 | T_2_-3-2 | 20230125 | T_2_-4-2 | 20230325 | T_2_-5-2 | 20230525 |
| T_2_-1-3 | 20221210 | T_2_-2-3 | 20221225 | T_2_-3-3 | 20230125 | T_2_-4-3 | 20230325 | T_2_-5-3 | 20230525 |
| T_3_-1-1 | 20221210 | T_3_-2-1 | 20221225 | T_3_-3-1 | 20230125 | T_3_-4-1 | 20230325 | T_3_-5-1 | 20230525 |
| T_3_-1-2 | 20221210 | T_3_-2-2 | 20221225 | T_3_-3-2 | 20230125 | T_3_-4-2 | 20230325 | T_3_-5-2 | 20230525 |
| T_3_-1-3 | 20221210 | T_3_-2-3 | 20221225 | T_3_-3-3 | 20230125 | T_3_-4-3 | 20230325 | T_3_-5-3 | 20230525 |
| T_4_-1-1 | 20221210 | T_4_-2-1 | 20221225 | T_4_-3-1 | 20230125 | T_4_-4-1 | 20230325 | T_4_-5-1 | 20230525 |
| T_4_-1-2 | 20221210 | T_4_-2-2 | 20221225 | T_4_-3-2 | 20230125 | T_4_-4-2 | 20230325 | T_4_-5-2 | 20230525 |
| T_4_-1-3 | 20221210 | T_4_-2-3 | 20221225 | T_4_-3-3 | 20230125 | T_4_-4-3 | 20230325 | T_4_-5-3 | 20230525 |
| CK-1-1 | 20221210 | CK-2-1 | 20221225 | CK-3-1 | 20230125 | CK-4-1 | 20230325 | CK-5-1 | 20230525 |
| CK-1-2 | 20221210 | CK-2-2 | 20221225 | CK-3-2 | 20230125 | CK-4-2 | 20230325 | CK-5-2 | 20230525 |
| CK-1-3 | 20221210 | CK-2-3 | 20221225 | CK-3-3 | 20230125 | CK-4-3 | 20230325 | CK-5-3 | 20230525 |

Table S2 Summary statistics of the defense hormone contents in xylem for *Aquilaria sinensis* under different induction treatments. JA: jasmonic acid, SA: salicylic acid, ACC: 1-amino-1-cyclopropanecarboxylic acid, ABA: abscisic acid. Values in table were mean ± standard error (n=15). The different letters in the same column show the significance at *p* < 0.05 between induction treatments.

| Induction treatment | Defense hormone content | | | |
| --- | --- | --- | --- | --- |
|  | JA (ng·g^-1^) | SA (μg·g^-1^) | ACC (μg·g^-1^) | ABA (ng·g^-1^) |
| CK | 49.81±0.30 e | 36.62±0.20 d | 4.67±0.03 d | 189.33±1.26 d |
| T_1_ | 67.96±2.17 b | 50.78±0.94 b | 7.29±0.23 c | 269.96±3.81 a |
| T_2_ | 66.06±1.41 c | 50.69±0.99 b | 7.60±0.26 b | 243.67±2.37 c |
| T_3_ | 65.37±1.40 d | 48.24±1.00 c | 7.22±0.25 c | 254.55±3.14 b |
| T_4_ | 72.15±2.32 a | 55.40±1.17 a | 7.94±0.21 a | 268.47±2.60 a |

Table S3 Summary statistics of the defense hormone contents in xylem for *Aquilaria sinensis* under different induction times. 15d, 30d, 60d, 120d and 180d represent 15^th^, 30^th^, 60^th^, 120^th^ and 180^th^ days after induction treatment.

| Induction time | Defense hormones content | | | |
| --- | --- | --- | --- | --- |
|  | JA (ng·g^-1^) | SA (μg·g^-1^) | ACC (μg·g^-1^) | ABA (ng·g^-1^) |
| 15d | 62.72±1.98 c | 47.78±1.66 c | 6.52±0.26 c | 249.70±9.56 a |
| 30d | 73.78±3.30 a | 53.35±2.33 a | 8.05±0.46 a | 238.74±6.89 b |
| 60d | 66.41±2.43 b | 49.13±1.75 b | 7.33±0.37 b | 247.53±7.65 a |
| 120d | 59.77±1.44 d | 46.76±1.52 c | 6.64±0.31 c | 240.73±7.42 b |
| 180d | 58.66±1.17 e | 44.70±1.38 d | 6.17±0.22 d | 249.28±9.51a |

Table S4 Comparisons of the growth hormone contents in xylem for *Aquilaria sinensis* among the induction treatments or induction times. GA: gibberellin; IAA: auxin.

| Induction treatment | Growth hormone content | | Induction  time | Growth hormone content | |
| --- | --- | --- | --- | --- | --- |
|  | GA (μg·g^-1^) | IAA (μg·g^-1^) |  | GA (μg·g^-1^) | IAA (μg·g^-1^) |
| CK | 10.83±0.24 a | 71.35±0.41 a | 15d | 7.24±0.47 c | 62.37±0.99 b |
| T_1_ | 6.51±0.18 d | 61.50±0.42 c | 30d | 7.44±0.36 c | 64.12±1.20 a |
| T_2_ | 7.27±0.13 c | 61.50±0.53 c | 60d | 8.01±0.41 b | 64.29±1.15 a |
| T_3_ | 8.07±0.11 b | 64.34±0.77 b | 120d | 8.43±0.48 ab | 65.17±1.07 a |
| T_4_ | 6.95±0.20 cd | 61.23±0.45 c | 180d | 8.52±0.40 a | 63.97±1.25 ab |

Table S5 Variations of the antioxidant enzyme activities in xylem for *Aquilaria sinensis* between different induction treatments. SOD: superoxide dismutase; CAT: catalase; POD: peroxidase. The different letters in the same row show the significance at *p* < 0.05 between induction treatments.

| Enzyme  activity | Induction treatment | | | | |
| --- | --- | --- | --- | --- | --- |
|  | CK | T_1_ | T_2_ | T_3_ | T_4_ |
| SOD (U·g^-1^) | 631.92±8.85 c | 939.47±35.91 a | 877.02±32.18 b | 848.72±22.33 b | 859.93±21.12 b |
| CAT (U·mg^-1^) | 20.75±0.44 d | 37.81±0.42 a | 36.07±0.58 b | 33.27±0.82 c | 37.82±0.60 a |
| POD (U·mg^-1^) | 19.12±0.29 d | 33.44±0.81 a | 27.81±0.57 c | 30.68±0.43 b | 30.15±0.52 b |

Table S6 Variations of the antioxidant enzyme activities in xylem for *Aquilaria sinensis* between different induction times

| Enzyme  activity | Induction time | | | | |
| --- | --- | --- | --- | --- | --- |
|  | 15d | 30d | 60d | 120d | 180d |
| SOD (U·g^-1^) | 780.50±26.77 c | 963.57±49.12 a | 853.88±36.64 b | 795.12±25.08 c | 764.00±16.24 c |
| CAT (U·mg^-1^) | 32.17±1.63 b | 35.84±1.96 a | 32.96±1.87 b | 32.48±1.74 b | 32.26±1.65 b |
| POD (U·mg^-1^) | 26.97±1.07 b | 29.81±1.73 a | 28.82±1.30 a | 28.68±1.44 a | 26.92±1.32 b |

Table S7 Comparisons of MDA and PRO contents in xylem for *Aquilaria sinensis* among the induction treatments or induction times

| Induction treatment | MDA (nmol·g^-1^) | PRO (μg·g^-1^) | Induction  time | MDA (nmol·g^-1^) | PRO (μg·g^-1^) |
| --- | --- | --- | --- | --- | --- |
| CK | 28.28±0.28 c | 413.08±3.76 c | 15d | 49.33±2.84 a | 592.58±26.03 c |
| T_1_ | 46.93±1.57 a | 655.37±24.91 b | 30d | 46.99±2.68 b | 715.57±40.12 a |
| T_2_ | 43.68±2.07 b | 641.80±22.14 b | 60d | 43.19±2.19 c | 629.79±28.67 b |
| T_3_ | 47.87±1.49 a | 638.32±21.78 b | 120d | 39.53±1.69 d | 578.51±22.46 c |
| T_4_ | 48.38±1.90 a | 676.35±23.29 a | 180d | 36.07±1.35 e | 508.48±15.38 d |

Table S8 Sugar contents in xylem for *Aquilaria sinensis* under different induction treatments or induction times

| Induction treatment | Sugar content (mg·g^-1^) | | Induction  time | Sugar content (mg·g^-1^) | |
| --- | --- | --- | --- | --- | --- |
|  | Total sugar | Soluble sugar |  | Total sugar | Soluble sugar |
| CK | 563.84±3.72 a | 84.98±0.74 a | 15d | 500.80±9.44 a | 71.40±1.94 b |
| T_1_ | 469.05±3.39 b | 69.74±1.07 b | 30d | 487.02±9.66 b | 68.75±2.36 c |
| T_2_ | 472.81±3.47 b | 69.35±1.31 b | 60d | 478.26±12.01 c | 71.65±1.64 b |
| T_3_ | 468.75±2.19 b | 69.02±1.30 b | 120d | 486.75±12.37 b | 69.81±2.14 bc |
| T_4_ | 467.27±2.59 b | 66.41±1.26 c | 180d | 488.89±7.77 b | 77.90±1.49 a |

Fig.S1 The GC-MS total ion chromatograms of agarwood samples


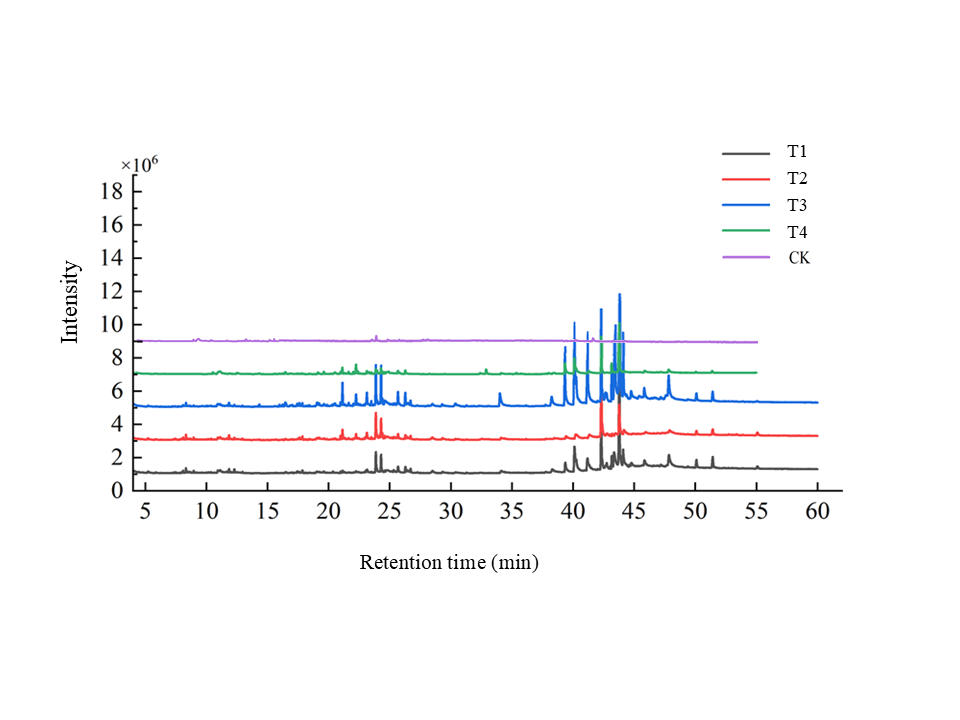

Supplement: Supplementary file 1 [file DataSheet1.docx]
